# Supplementary material for: Association of APOE e2 genotype with Alzheimer’s and non-Alzheimer’s neurodegenerative pathologies
Source: Nat Commun. 2020 Sep 18;11:4727. doi: 10.1038/s41467-020-18198-x (PMC7501268; doi:10.1038/s41467-020-18198-x)
Supplement: Supplementary file 1 — Supplementary Information [file 41467_2020_18198_MOESM1_ESM.pdf]

Association of APOE e2 Genotype with Alzheimer's and Non-Alzheimer's Neurodegenerative Pathologies

Goldberg et al.

Supplementary Information

Supplementary Table 1
APOE Genotype by PLAQUE THAL STAGE

Frequency	APOE Genotype	NPTHAL(NPTHAL) 0	1	2	3	4	5	Total
	**E2**	48	14	8	17	16	27	130
	**E3/E3**	133	75	60	90	145	250	753
	**E3/E4**	14	25	25	61	109	301	535
	**E4/E4**	3	1	3	5	20	107	139
	**Total**	198	115	96	173	290	685	1557

Statistics for Table 1: APOE Genotype by NPTHAL

Statistic	DF	Value	Prob
**Chi-Square**	15	263.2261	<.0001

Supplementary Table 2
APOE Genotype by BRAAK STAGE

	APOE Genotype	BRAAK STAGE							
		**0**	**1**	**2**	**3**	**4**	**5**	**6**	**Total**
**Frequency**	**E2**	19	17	15	27	15	12	25	130
	**E3/E3**	53	78	100	84	94	130	214	753
	**E3/E4**	11	28	22	44	52	112	266	535
	**E4/E4**	0	2	2	2	6	30	97	139
	**Total**	83	125	139	157	167	284	602	1557

Statistics for Table 2: APOE Genotype by NACC BRAAK STAGE

Statistic	DF	Value	Prob
**Chi-Square**	18	234.6745	<.0001

Supplementary Table 3
APOE Genotype by NEURITIC PLAQUE FREQUENCY (# cases)

Frequency	APOE Genotype	NEURITIC PLAQUE STAGE				
		**0**	**1**	**2**	**3**	**Total**
	**E2**	61	13	19	37	130
	**E3/E3**	231	78	154	290	753
	**E3/E4**	51	34	108	342	535
	**E4/E4**	5	5	20	109	139
	**Total**	348	130	301	778	1557

Statistics for Table 3: APOE Genotype by NEURITIC PLAQUE

Statistic	DF	Value	Prob
**Chi-Square**	9	209.1753	<.0001

Supplementary Table 4. Independent Age and Sex Effects on AD Neuropathologies

		Wald	p	Point Estimate	CI
**Braak**	Age	2.39	.12	.99	.98 – 1.00
**e2 v e3**	Sex	5.59	.02	.75	.59 – .95
**Braak**	Age	5.57	.02	1.02	1.00 – 1.03
**e2 v e4**	Sex	4.12	.04	.76	.58 – .99
**Thal**	Age	1.09	.29	.99	.99 – 1.01
**e2 v e3**	Sex	4.08	.04	.78	.62 – .99
**Thal**	Age	.17	.67	.99	.98 – 1.01
**e2 v e4**	Sex	4.51	.03	.76	.39 – .77
**Neuritic Plaque**	Age	.17	.68	1.00	.59 – 1.01
**e2 v e3**	Sex	4.50	.03	.77	.60 – .98
**Neuritic Plaque**	Age	9.51	.002	1.02	1.01 – 1.04
**e2 v e4**	Sex	2.63	.10	.79	.59 – 1.05

Supplementary Table 5
APOE e2/e4 by BRAAK STAGE

Frequency	APOE	BRAAK STAGE							
		**0**	**1**	**2**	**3**	**4**	**5**	**6**	**Total**
	**E2/E3**	19	17	15	26	15	12	23	127
	**E2/E4**	0	0	5	3	4	12	22	46
	**E3/E3**	52	77	99	84	92	128	210	742
	**E3/E4**	11	28	22	41	52	106	258	518
	**Total**	82	122	141	154	163	258	513	1433

Statistics for Table 5: APOE e2/e4 by NACCBRAA

Statistic	DF	Value	Prob
**Chi-Square**	18	157.7241	<.0001

Supplementary Table 6
APOE e2/e4 by NEURITIC PLAQUE

Frequency		NEURITIC PLAQUE				
		**0**	**1**	**2**	**3**	**Total**
	**E2/E3**	61	13	18	35	127
	**E2/E4**	3	4	11	28	46
	**E3/E3**	228	78	150	286	742
	**E3/E4**	51	34	106	327	518
	**Total**	343	129	285	676	1433

Statistics for Table 6: APOE e2/e4 by NEURITIC PLAQUE

Statistic	DF	Value	Prob
**Chi-Square**	9	153.8538	<.0001

Supplementary Table 7. APOE e2/e4 by THAL AMYLOID

Frequency	APOE	0	1	2	3	4	5	Total
	E2/E3	48	13	8	17	16	25	127
	E2/E4	2	2	3	3	13	23	46
	E3/E3	131	74	60	90	141	246	742
	E3/E4	14	25	25	60	103	291	518
	Total	195	114	96	170	273	585	1433

Statistics for Table 7: APOE e2/e4 by THAL

Statistic	DF	Value	Prob
Chi-Square	15	190.2329	<.0001

Supplementary Table 8
APOE Genotype by LEWY BODY EXTENT

Frequency	APOE Genotype	LEWY)				
		0	1	2	3	Total
	E2	88	15	21	9	133
	E3/E3	543	30	121	78	772
	E3/E4	319	15	120	81	535
	E4/E4	65	2	45	30	142
	Total	1015	62	307	198	1582

Statistics for Table 8: APOE Genotype by LEWY BODY EXTENT

Statistic	DF	Value	Prob
Chi-Square	9	74.6915	<.0001

Supplementary Table 9. Age and Sex Effects on Lewy Body Pathology

		**Wald**	**p**	**Point Est.**	**CI**
**Lewy Body**	Age	.09	.77	1.00	.99 – 1.01
**e2 v e3**	Sex	3.52	.06	.76	.57 – 1.01
	Age	2.74	.10	.99	.98 – 1.00
**e2 v e4**	Sex	.25	.61	.93	.70 – 1.23

Supplementary Table 10. APOE by TDP-43

	APOE	TDP-43		
		**0**	**1**	**Total**
**Frequency**	**E2**	90	17	107
**Percent**		7.20	1.36	8.56
**Row Pct**		84.11	15.89	
**Col Pct**		7.85	16.50	
	**E3/E3**	550	54	604
		44.00	4.32	48.32
		91.06	8.94	
		47.95	52.43	
	**E3/E4**	404	27	431
		32.32	2.16	34.48
		93.74	6.26	
		35.22	26.21	
	**E4/E4**	103	5	108
		8.24	0.40	8.64
		95.37	4.63	
		8.98	4.85	
	**Total**	1147	103	1250
		91.76	8.24	100.00

Frequency Missing = 227

Supplementary Table 11. APOE by PSP

	APOE	PSP		
		**0**	**1**	**Total**
**Frequency**	**E2**	97	10	107
**Percent**		7.76	0.80	8.56
**Row Pct**		90.65	9.35	
**Col Pct**		8.09	19.61	
	**E3/E3**	576	28	604
		46.08	2.24	48.32
		95.36	4.64	
		48.04	54.90	
	**E3/E4**	421	10	431
		33.68	0.80	34.48
		97.68	2.32	
		35.11	19.61	
	**E4/E4**	105	3	108
		8.40	0.24	8.64
		97.22	2.78	
		8.76	5.88	
	**Total**	1199	51	1250
		95.92	4.08	100.00

Frequency Missing = 227

Supplementary Table 12. APOE by Pick's B0dy Pathology

	APOE	Pick		
		0	1	Total
Frequency<br>Percent<br>Row Pct<br>Col Pct	E2	103<br>8.24<br>96.26<br>8.41	4<br>0.32<br>3.74<br>16.00	107<br>8.56
	E3/E3	586<br>46.88<br>97.02<br>47.84	18<br>1.44<br>2.98<br>72.00	604<br>48.32
	E3/E4	428<br>34.24<br>99.30<br>34.94	3<br>0.24<br>0.70<br>12.00	431<br>34.48
	E4/E4	108<br>8.64<br>100.00<br>8.82	0<br>0.00<br>0.00<br>0.00	108<br>8.64
	Total	1225<br>98.00	25<br>2.00	1250<br>100.00

Frequency Missing = 227
